# A School-Based Progressive Muscle Relaxation Program for Female Adolescents: Development and the Effectiveness on Physiological and Psychological Evidence

**DOI:** 10.3390/healthcare9101319

**Published:** 2021-10-03

**Authors:** Mei-Li Tsai, Tsan-Hwang Cheng, Yen-Kuang Yang, Chi-Jane Wang

**Affiliations:** 1 Department of Nursing, Chung Hwa University of Medical Technology, Tainan 71703, Taiwan; 03608@ms.hwai.edu.tw; 2 Department of Nursing, College of Medicine, National Cheng Kung University, Tainan 70101, Taiwan; 3 Department of Pharmaceutical Science and Technology, Chung Hwa University of Medical Technology, Tainan 71703, Taiwan; wawa@mail.hwai.edu.tw; 4 Department of Medicine, College of Medicine, National Cheng Kung University, Tainan 70101, Taiwan; ykyang@mail.ncku.edu.tw

**Keywords:** adolescent, stress, physiology, muscle relaxation, hydrocortisone, homeostasis

## Abstract

(1) Background: A variety of stressors may be potentially harmful to adolescents’ health and well-being. Relaxation techniques have been recognized as a valid method for stress release, but the challenge is to apply them practically in schools to produce the desired effects. (2) Methods: This feasibility study used the Perceived Stress Scale (PSS) and hair cortisol concentration (HCC) to test the effects of an abbreviated progressive muscle relaxation (APMR) program on female adolescents. The participants were recruited from a high school and assigned by class cluster to either the experimental group (EG, *n* = 40) or the control group (CG, *n* = 35). Both received 4 weeks of stress-related lessons. The EG received 60 additional sessions of APMR over 12 weeks. (3) Results: The program dropout rate of the participants was 1.3%. The EG’s program adhesion rate was 99.1%, and nearly half felt satisfied with the program. After adjusting for the BMI and the pretest in the ANCOVA, it was found that the CG had a greater change in HCC between the pre- and post-tests than the EG, while the PSS did not change significantly in either group. (4) Conclusion: APMR is a valid practice for physiological homeostasis of HCC for female adolescents, but it has no significant effect on perceived stress.

## 1. Introduction

Adolescents often experience various kinds of pressure that come from the academic performance expectations of their parents or themselves or from emotional and interpersonal conflicts [1,2]. Academic performance is a major stressor among adolescents worldwide, especially in eastern countries [3]. Chronic and persistent stress can lead to many adverse health problems, including physical illness [4], mental and social problems, or health-risk behaviors [3,5,6]. Environmental stressors have long-lasting effects on the development of humans; therefore, adolescence is perceived as a period involving relatively more challenges than other age stages [7]. A lack of awareness of appropriate strategies to deal with stressful experiences can put adolescents at risk of poor coping, such as emotional maladjustment [8].

Many relaxation techniques have proven to be effective and are used by young people to relieve stress, such as progressive muscle relaxation (PMR) [9], meditation [10], and mindfulness-based stress reduction [11]. A systematic review and meta-analysis research on adolescents’ use of these relaxation strategies has concluded that practicing relaxation techniques under supervision is significantly more effective than doing so without supervision or merely receiving psychoeducation [12]. Practicing mindfulness-based stress reduction and meditation may be potentially harmful to individuals, even when implemented under correct guidance. Participants may experience post-intervention anxiety or negative effects during the intervention period [13]. PMR was adapted from Jacobson’s relaxation technique [14]. The muscle groups involved in PMR variations range from 7 to 16. The seven-muscle group abbreviated progressive muscle relaxation (APMR) technique is time-efficient and requires fewer sessions to complete training [15]. APMR may be preferred in schools because it is a simpler and more cost-effective technique including progressive contracting and releasing of seven major muscle groups of the human body [15,16]. Thus, APMR can be used as a body–mind intervention for adolescents.

Both subjective and objective stress level measurements are frequently used to evaluate the effectiveness of relaxation techniques [9,17,18]. The self-report questionnaires for subjective measurements include the Perceived Stress Scale (PSS) [18], Stress-O-Meter [18], and emotion-related scales [17,19]. Objective measurements of physiological indicators include heart rate and variability, blood pressure, saliva, and serum cortisol concentration [9,17,18,19].

It is well-known that stress is a behavioral and physiological response to stressors [18]. The most commonly discussed physiological stress response is the hypothalamus-pituitary–adrenal (HPA) axis, which mediates the cross-talk between the brain and body [20]. It is the main neuroendocrine axis that regulates homeostasis in human beings [21]. Cortisol is an important mediating hormone that is rapidly synthesized and secreted from the adrenal gland in response to stress. Adolescents are highly vulnerable to stress, probably because of their protracted cortisol stress response, which persists into adulthood [22]. Several studies have revealed that both hyper- and hypo-cortisol can have harmful health consequences [23,24].

Saliva or serum cortisol can only measure the short-term effects of stress-related outcomes [25,26]. Hair cortisol concentration (HCC) is a potentially reliable endogenous biomarker for the retrospective assessment of long-term central HPA axis activity and is considered a non-invasive measurement of stress response in adolescents [27]. HCC can be used to evaluate the effects of interventions on the biological signature of stress [28,29]. Even though the participants’ characteristics and research purposes varied across previous studies, the findings verified that the stabilization of cortisol levels is beneficial for improving psychosocial well-being [28]. In addition, researchers have found that either PMR or APMR intervention could decrease saliva cortisol, but there is a lack of evidence of PMR intervention’s effect on adolescent HCC [18,19].

In previous studies, the duration of PMR interventions for adolescents ranged from a single dose to 8 weeks, and each session typically lasted for 15 to 45 min, two to five times per week [9]. Regarding the guidelines and place for PMR implementation, some studies allowed students to practice daily at home after they had been trained once or twice by a specialist [9,17,18,19]. The implementation of relaxation techniques at home cannot guarantee that the participants would fully comply with the intervention guidelines; as such, the program’s effectiveness could have potentially been underestimated. To avoid similar issues, the present study carefully considered such factors while determining the study design, program duration, session length, and place of practice. A feasibility study revealed that school-based interventions supervised by school nurses can optimize the interest level in the intervention and minimize the participation barriers [30]. Accordingly, it is valuable to design a body–mind relaxation program to directly release muscle tension and indirectly maintain homeostasis regulation in adolescents.

The purposes of this study were to test the feasibility of a 16-week school-based APMR program among female adolescents and to conduct a preliminary evaluation of its effects on the subjective and objective aspects of stress relaxation. Consequently, the following hypotheses were proposed: (1) school-based APMR is a feasible and acceptable relaxation technique for female adolescents; (2) the HCC in the experimental group (EG) will not change significantly (implying physiological homeostasis) before and after the APMR intervention, whereas the control group (CG) will; (3) the perceived stress will change significantly for the EG after the APMR intervention, but not for the CG.

## 2. Materials and Methods

### 2.1. Design

This study employed a quasi-experimental design with a pre-test and post-test to evaluate the feasibility and preliminary effects of a school-based APMR program for female adolescent students.

### 2.2. Setting and Sample

In the present feasibility study, a convenience sample of adolescents was recruited from a high school in southern Taiwan that had mostly female students. First, the researchers visited the principal to explain the research process and obtain approval for the APMR program.

For the study, the participant inclusive criterion was having a hair length of more than 3 cm on the posterior vertex position of the head. The exclusion criteria were taking any corticosteroid medication within the past 3 months, applying hair dye or perm within the past 3 months, and/or experiencing major life events (the death or serious illness of a close relative or parents’ divorce) [31] within the past 6 months. Thereafter, the subjects were assigned to either the EG or CG.

### 2.3. Ethical Considerations

This study was approved by the Ethics Committee of Kaohsiung Armed Forces General Hospital (approval no. KAFGH106-035). The research details were shared with the parents through a consent letter. The researcher explained the same information to the students from two classes during a classroom visit. All the participants and their parents provided written informed consent before participating in the study.

### 2.4. Partnership Team for Program Development

The program’s objectives, approaches, protocols, and implementation process were developed by the partnership team consisting of the principal, deans, department heads, health promotion professionals, and school nurses. A school-wide assessment of the intervention was conducted subsequently.

At least six team meetings were held to facilitate the development and implementation of the APMR program. In these meetings, the team discussed the intervention protocols for the actual course, analyzed the need for an intervention space, planned the course progression, and designed the methods to assist and monitor the participants in the intervention space. The intervention protocol was regularly modified and adjusted in close collaboration with the members of the school staff. The teachers for the two classes and the school nurses received a 2 h training course before starting the implementation to solidify their stress-related knowledge and skills, which were required to supervise the APMR practice during the intervention period.

### 2.5. The School-Based APMR Program

A 16-week APMR program was designed to help the students build stress-related knowledge and learn the APMR techniques to be practiced according to an assigned schedule.

#### 2.5.1. Stress-Related Knowledge Education

The lessons comprised four educational talks over four weeks, including topics focusing on the “concept of stress and stressors”, “biological process of stress”, “relationship between stress and physical and psychological health”, and “health benefits of APMR”. These were held as part of a health-related elective course between the 3rd and the 6th week of the semester. Both the EG and CG received these educational talks, but the EG received one additional class that introduced APMR. The program also provided the activity tutors with a 10 min CD as an assistive audio script to guide the implementation over the next 12 weeks to help ensure the quality of the APMR practice.

#### 2.5.2. Techniques for APMR Practice

Considering the limited time and the need to guarantee that the students’ formal curriculum and class time were not disrupted, the two 10 min practice sessions of APMR techniques were arranged during break times (during the morning break and after the noon nap). We treated academic exams as a significant real-time stressor for adolescents. Therefore, the practice sessions took place for 10 days before each monthly exam or professional certification exam, resulting in a total of 30 days of practice over 12 weeks.

**Figure 1:**
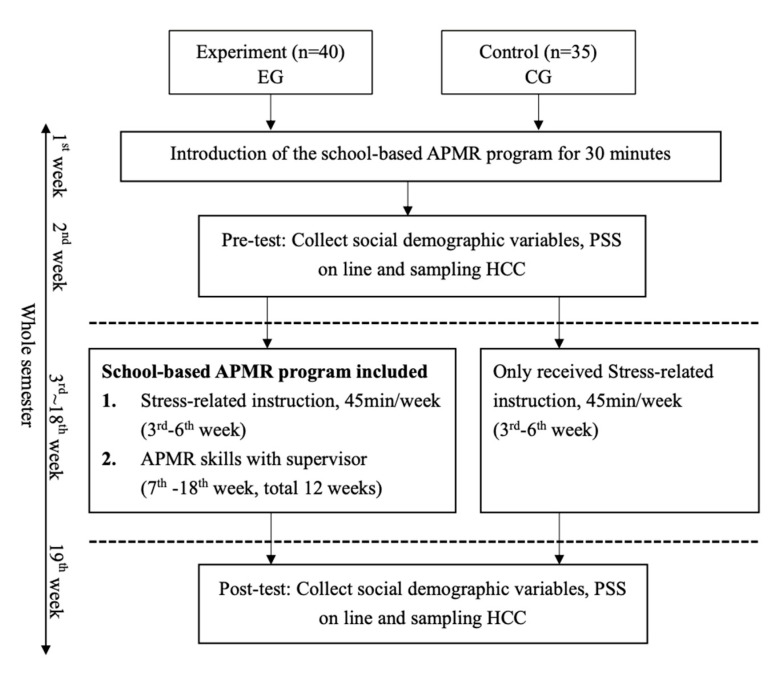
Experimental process of the study.

The APMR sessions were conducted by an expert and university trainer during the 6th week of this program. The muscle relaxation technique targeted the following major muscle groups: hands, arms, neck, shoulders, feet, and legs. Those in the EG were encouraged to focus their sensations on the release of muscle tension and the feeling of comfort. They were advised not to tense the muscle groups when they felt strained or aggravated pain. They learned about the steps of APMR: (a) secure a quiet environment in the classroom (this was carried out by the tutors); (b) assume a comfortable sitting position to eliminate muscular tension; (c) systematic tense up the muscles for 5 s, then relax the muscles for 10 s progressively; (d) focus on the comfortable and relaxed feeling as the muscles relax [14,18]. Each practice session used the audio script from the CD and was supervised by a tutor. Figure 1 shows the recruitment of participants and the implementation of the school-based APMR program during the semester.

### 2.6. Evaluation of Program Feasibility

The indicators of primary outcomes, including the program dropout rate, adherence, and activity satisfaction, were collected to assess the study’s feasibility [32,33,34]. All recruitment efforts were tracked, and the researchers recorded the number of participants who were recruited and screened as per the inclusion and exclusion criteria. The reasons for ineligibility or refusal were also recorded. The APMR protocol was recorded to understand the needs of the tutors and participants in the process.

In the present feasibility study, each group’s dropout rate was calculated by dividing the number of participants who quit the entire program by the total number of participants. Adherence to participation in the EG was assessed using the Person-Time Follow-Up Rate (PTFR), dividing the observed person-time by total person-time, excluding the dropouts. The EG participants were asked to rank their overall satisfaction with the APMR program from 0 (not at all satisfied) to 4 (very satisfied). In addition, they were welcome to submit any comments or needs during the intervention.

The primary outcome—feasibility—was defined as achieving the target sample size of at least 20 participants in each group, <10% dropout rate, >90% adherence (also called PTFR) to the school-based APMR program, and >75% of participants being “satisfied” or “very satisfied” with the program. If the indicators failed to achieve these feasibility goals, a discussion and modification should be conducted for the research design, protocol, and intervention before conducting a randomized controlled trial in the future.

### 2.7. Evaluation of APMR Effects

#### 2.7.1. Perceived Stress Scale

The PSS is the most frequently used psychological instrument for measuring the perception of stress and is suitable for respondents with at least a junior high school education. The Chinese version of the PSS consists of seven positive and seven negative items. The participants were asked to rate each item’s occurrence frequency during the past month on a 5-point Likert scale ranging from 0 (never) to 4 (routinely). Higher scores indicate higher stress in the respondents’ daily lives. A confirmatory factor analysis corroborated the two-factor structure of the model. The positive and negative factors correlated significantly and negatively to a moderate extent. The instrument also had a high internal consistency reliability (Cronbach’s alpha > 0.85) [35].

#### 2.7.2. Visual Analog Scales of Relaxation Rating

Previous studies have demonstrated that visual analog scales (VAS) have adequate psychometric properties to measure acute pain or level of relaxation (Cronbach’s alpha > 0.80) [36,37]. In the present study, a VAS was used for a subjective relaxation rating to calculate the participants’ attendance for adherence. Before and after each APMR practice session, the participants were asked to rate their level of relaxation on a scale from 0 (not relaxed) to 6 (highly relaxed). These self-reported data were collected via a paper-and-pencil administration of the instrument.

#### 2.7.3. Hair Cortisol Sample Collection and Analysis

HCC is a stress indicator and mental health-related variable that can be significantly influenced by age, sex, hair treatment, body mass index (BMI), and major life events [24,31]. Body weight, body height, and BMI (kg/m^2^) were calculated using anthropometric measurements after calibrating the equipment. The participants were asked to provide two 3 cm hair samples from different locations of their scalp. The research assistant was trained to cut 20–30 hair strands from the scalp’s vertex posterior, as close to the scalp as possible, using scissors. The most proximal 3 cm segment reflects systematic cortisol exposure over the previous 3 months. The level of HCC was measured according to the modified hair cortisol analysis protocol previously reported and analyzed on a salivary ELISA kit manufactured by Alpco Diagnostics for quantification [38,39,40,41].

### 2.8. Data Analysis

Frequencies and percentages were used to describe the participants’ body weight, body height, and BMI characteristics, and an independent *t*-test compared these characteristics between the EG and CG. To reduce the skewness to approximately twice the standard error or less, the HCC data used a log transformation to perform further analysis [28]. A paired sample *t*-test and ANCOVA were used to evaluate the effectiveness of the outcomes. Statistical analyses were performed using the statistical software package SPSS 24.0 for Windows.

## 3. Results

**Figure 2:**
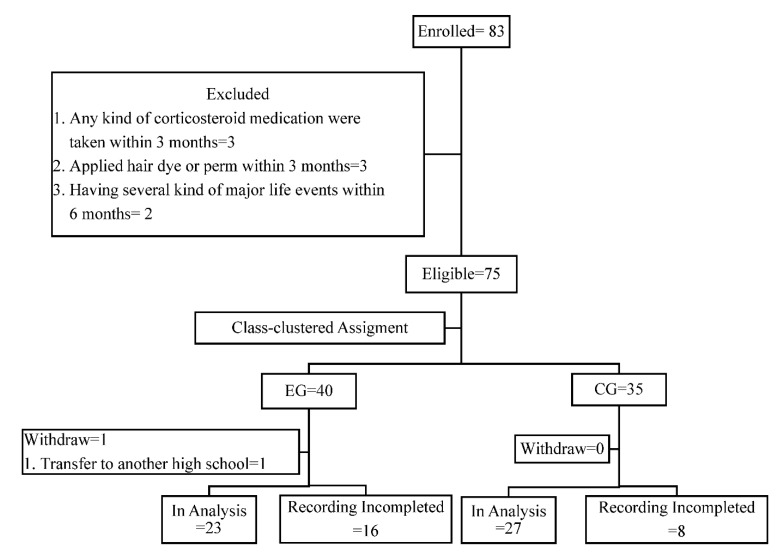
Flowchart of the study participants recruitment.

The target sample size was obtained in 2 weeks. Figure 2 presents the procedures involved in participant recruitment. A total of 75 eligible female adolescents in the 11th and 12th grades (16–18 years of age) were recruited from the 83 female students in the two classes. They were assigned to the EG (*n* = 40) and CG (*n* = 35). The two classes were located in two different buildings on the campus to avoid information contamination. The remaining eight students were excluded as they did not meet the inclusion criteria. Finally, the complete data from 23 participants in the EG (response rate: 60%) and 27 in the CG (response rate: 77.1%) were included in the analysis.

Seventy-four participants completed the research activities, and only one participant dropped out (1.3%) due to school transfer. The adherence (PTFR) was 99.1% for the school-based APMR program. Only one of the remaining 39 participants failed to complete the program activities, as she went on personal leave and missed 20 practice sessions. Nearly half (46.5%) of the participants reported that they were “satisfied” with this program. Some of the participants verbally stated that they felt relaxed after performing APMR.

There were no significant differences in the baseline characteristics between the two groups (Table 1). After adjusting for the BMI and pre-test scores in the ANCOVA, the changes in the PSS scores were not significantly different between the two groups before and after the APMR intervention (*p* = 0.59). However, changes in HCC were significantly greater in the CG (*p* = 0.02). There was a significantly larger HCC change in the within-group CG analysis (*p* = 0.001), but not in the EG. Additionally, the VAS revealed a significant increase in the relaxation rating of the EG (*p* < 0.001) (Table 2).

## 4. Discussion

The purposes of the present study were to test the feasibility of the school-based APMR program and to conduct a preliminary evaluation of the subjective and objective aspects of stress relaxation among female adolescents over a semester. Contrasting the results to the study’s a priori definitions of feasibility, we found that the dropout rate (1.3%) was lower than 10%, and the adherence to the program (PTFR; 99.1%) was greater than 90%. However, a low satisfaction rate of 46.2% in the EG fell short of the goal of 75% satisfaction rate. Thus, while several indicators suggested that the program was feasible, there are still issues that require further investigation.

A prior feasibility study of a 14-week school-based coping intervention with 2 h weekly sessions supervised by school nurses had a dropout rate, adherence, and response rate that were similar to ours, but with a higher satisfaction rate [30]. Regarding the low satisfaction rate for our APMR program, some participants complained that the second practice session, which occurred after their afternoon nap, was not suitable as they were already relaxed then. This may explain why the satisfaction rate was not over 50%, and the students were merely “satisfied” with the program. The participants suggested that future APMR could be implemented during the break after a few afternoon classes.

While the target sample size was obtained, the response rate was low at 60% for the EG and 77.1% for the CG. This raised some questions about the school-based APMR program’s acceptability as a treatment option for this population. The current study’s low response rate is similar to what was observed in past studies [30,42]. Nearly all the adolescents attended every practice session, but fewer completed the questionnaires online, despite considerable efforts to improve their completion rate without pressure. This indicates that students may have low interest in the research aspect of the APMR program but high adherence to the APMR itself.

Finally, we conclude that even though the satisfaction rate was low, having clear time restrictions, low time commitment, and practice with supervisors promote compliance and facilitate the program’s integration into the school environment without disrupting the students’ formal curriculum. Moreover, as educators are key partners who have a close relationship with adolescents, they are more suitable to deliver a stress reduction program and practice to promote mental health for adolescents [30].

The HCC results showed a more significant change (*p* = 0.02) in the CG, but there was no difference between the two groups in the PSS. Additionally, the VAS of relaxation rating showed a significant improvement for the EG (*p* < 0.001), implying that practicing with supervisors can ensure fidelity in the implementation of APMR. Furthermore, HCC results were reliable owing to the compliance of APMR and a steadier state in the EG during the school-based APMR program.

Pawlow and Jones [18] claimed that PSS scores decreased after a single 20 min dose of APMR, but it is difficult to compare perceived stress in the context of a short intervention. One study reported that after an 8-week stress management program including diaphragmatic breathing and progressive muscles relaxation, there was no difference in the PSS scores between the EG and the CG, even though there was a statistically significant decrease in the self-reported stress level in both groups [42]. Similarly, in the current study, after a 12-week school-based APMR program under the tutor’s guidance, the intervention did not affect the PSS. Notably, the VAS results on self-rated relaxation in the present study showed that the EG became significantly more relaxed after the APMR intervention [18,42], but such relaxation was not reflected in the PSS scores. A possible explanation is that the participants reported low stress levels on the PSS initially, and so a small post-intervention change would be difficult to detect in the post-test. Additionally, most participants completed the VAS and significantly improved their daily relaxation level, but the PSS had low response rates, which could have influenced the results due to lower inference validity [43]. Therefore, we infer that the data may have underestimated the effectiveness of the school-based APMR program.

HCC is a long-lasting accumulated biomarker that is more suitable for tracking long-term stress responses [44]. The HCC analysis showed a significant change between the pre- and post-test in the CG (*p* = 0.002) but not in the EG, implying that EG was in a steadier state throughout the APMR intervention in the school. A previous randomized control trial that evaluated the effect of an 8-week humanitarian intervention for war-affected adolescents showed that, regardless of whether one group had hyper- or hypo-secretion in HCC, the intervention could normalize HCC levels in either direction (up- or down-regulation) [28]. In that research, the participants comprised war-affected adolescents who experienced early life stress that could lead to dysregulation of cortisol signaling [28,45]. Thus, the effect of within-group differences in HCC could be detected after an intervention.

In contrast, the subjects in the present study were healthy female adolescents who had not suffered any major early life stress events. Thus, the difference between these target populations results in a lack of benchmarks against which we can compare the results. Finally, self-regulation and normalization of HCC that are consistent with the hypothesis of physiological homeostasis have been proposed previously [28]. The present study’s results also led to a similar conclusion; that is, the EG was in a steady state during the 16-week APMR period because there was no significant change in the HCC in the EG compared with the CG.

Based on the mechanism of physiological homeostasis [45,46,47,48], cortisol acts as a diverse array of physiological effects on the regulation of metabolic processes, immune system, reproduction, cognitive functions, and behaviors to prepare our body to cope with and recover from the stressors [49]. It is essential to maintain homeostasis of the cortisol level for body–mind health. In the present study, the HCC change between the pre- and post-tests was smaller in the EG, indicating that the cortisol level was more stable in the EG adolescents and more in conformity with the steady state. Therefore, the school-based APMR program is determined as appropriate for regulating the homeostasis of HCC in the long term.

In addition, a significant difference between the pre- and post-test results of HCC in the CG is required to determine APMR’s potential influence in the present study. A longitudinal study examined the levels of HCC when children transitioned into a more formal learning setting. The findings indicate that a larger increase in HCC is significantly associated with lower behavioral inhibition, which is the ability to regulate and control behavioral impulses [29]. Adolescence is commonly characterized by behavioral impulsivity. Further research is needed to explore whether the dysregulation of HCC in school-going adolescents is related to their behavioral inhibition ability.

There are several limitations to the present study. First, the target population was female high school adolescents, and thus, the findings cannot be generalized to other populations. Second, only 60% of the EG and 77.1% of the CG completed the PSS; such low response rates could have critically affected the study results. Third, several recent interventional studies have found that PMR could reduce saliva cortisol levels after the intervention [18,19,50]. HCC is significantly correlated with integrated salivary cortisol levels measured repeatedly over a month, and increasingly longer (at least 3 weeks) accumulation time yields increasingly stronger correlations between the two [40]. In fact, most of the current PMR control trials measure the saliva cortisol level 1 to 5 days per week for the pre- and post-evaluation period (short-term accumulation time), but the HCC can be used to evaluate the long-term retrospective effects for 1–3 months. Therefore, it is difficult to discuss the present study’s findings in light of saliva cortisol levels, as the two are not quite comparable.

## 5. Conclusions

In the present study, a significantly greater change in HCC was observed between the pre- and post-tests in the CG, while the PSS scores were not significantly different between the two groups. The school-based APMR program can be considered a potential relaxation technique to maintain the steady state for female adolescents, but it should utilize an audio script device to ensure adherence to all activities under tutor supervision.

For future randomized controlled trials, this school-based APMR study suggests the following: (1) the duration of the program should be expanded; (2) the second practice session should be scheduled on the days when the break time is scheduled in the afternoon; (3) the outcome indicators should be repeatedly measured during the implementation period and the behavioral inhibition indicators should be considered; (4) the program should be expanded to other schools, different ethnic populations, and age groups; and (5) the research should further explore the possible reasons for HCC dysregulation among school adolescents.

## Figures and Tables

**Table 1:** Comparisons of basic characteristics for two groups.

Variables	Control Group (*n* = 27)	Experimental Group (*n* = 23)
	Mean (SD)	
Body Height (cm)	159.8 (5.7)	162.5 (5.4)
Body Weight (kg)	55.9 (11.3)	56 (10.5)
Body Mass Index (kg/m^2^)	21.8 (3.9)	21.2 (3.7)
Measurements		
Perceived Stress Scale	29.3 (7.4)	27.9 (6.1)
Log Hair Cortisol Concentration (pg/mg)	1.9 (0.5)	1.8 (0.5)

Significances were tested using independent *t*-tests.

**Table 2:** The means of percentages, standard deviations, *p* values, and Partial Eta Squared for PSS and log hair cortisol concentration by ANCOVA.

Variables	Groups	Mean (95% Confidence Interval)		Within *p* Value	Between *p* Value	Partial Eta Squared
		Pre-Test	Post-Test			
Perceived Stress Scale	E	27.9 (15.9~39.8)	25.1 (5.4~44.8)	0.91	0.59	0.01
	C	29.3 (14.7~43.9)	27.6 (9.3~45.8)	0.22		
Log Hair Cortisol Concentration	E	1.9 (0.9~2.8)	1.8 (0.9~2.7)	0.64	0.02 *	0.12
	C	1.7 (0.7~2.7)	1.4 (0.2~2.5)	0.001 ***		
Visual Analog Scales of Relaxation Rating	E	2.1 (0.6~3.6)	3.3 (1.5~3.3)	<0.001 ***		

*** *p* < 0.001, * *p* < 0.05; statistical significance tests were paired *t*-tests for within-group and ANCOVA for between-group.

## Data Availability

The data presented in this study are available on request from the corresponding author. The data are not publicly available due to ethical reason.
